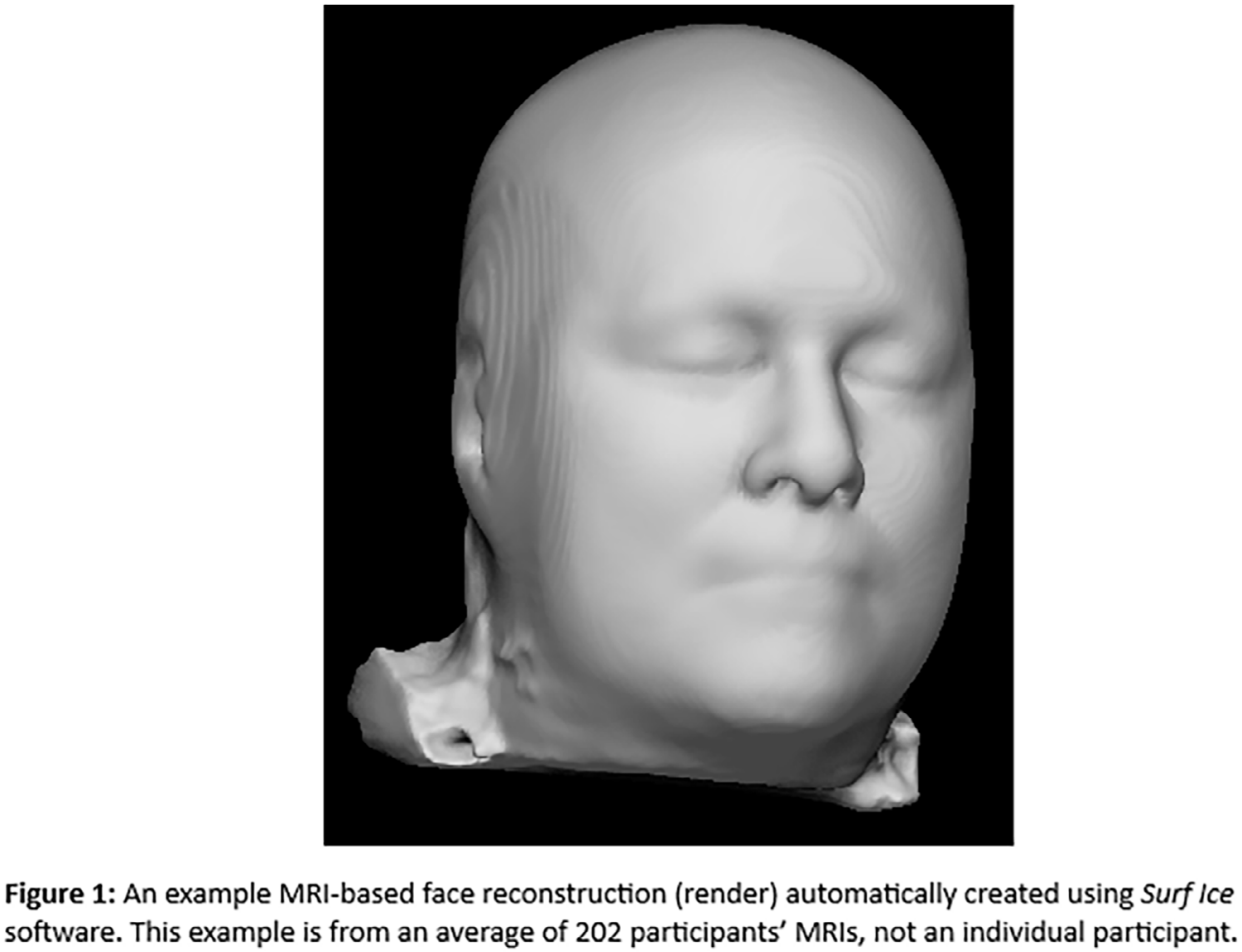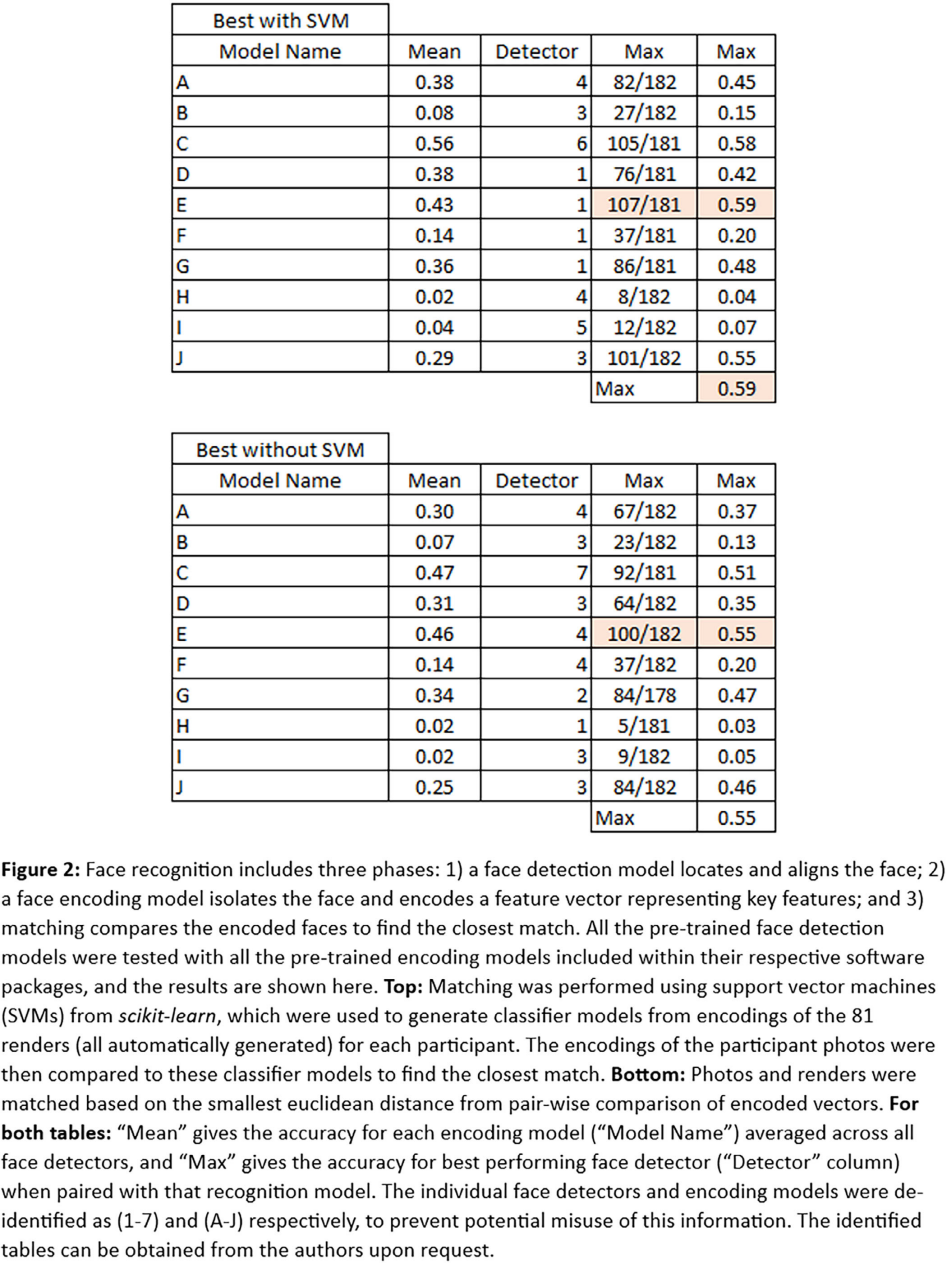# Measuring the Potential Risk of Re‐identification of Imaging Research Participants from Open‐Source Automated Face Recognition Software

**DOI:** 10.1002/alz.091462

**Published:** 2025-01-09

**Authors:** Carl M. Prakaashana, Marios Savvides, Jeffrey L. Gunter, Matthew L. Senjem, Prashanthi Vemuri, Kejal Kantarci, Jonathan Graff‐Radford, David S. Knopman, Ronald C. Petersen, Clifford R. Jack, Christopher G. Schwarz

**Affiliations:** ^1^ Mayo Clinic, Rochester, MN USA; ^2^ Carnegie Mellon University, Pittsburgh, PA USA; ^3^ Department of Radiology, Mayo Clinic, Rochester, MN USA

## Abstract

**Background:**

Research subjects can potentially be re‐identified from de‐identified MRI, CT, and PET brain scans with up to 98% accuracy using Microsoft Azure’s cloud‐based commercial facial recognition software. This showed the need to “de‐face” publicly shared research brain scans. Subsequently, Microsoft has begun restricting its face recognition services, intending to prevent misuse. This study tests a variety of popular open‐source computer vision and facial recognition software packages to measure the re‐identification risk with only unrestricted free software.

**Method:**

This study used brain MRI from Siemens 3T scanners and face photographs from 182 participants (ages 30‐90+) in the Mayo Clinic Study of Aging. Each participant had photos taken with an iPad, which were cropped loosely around the head and converted to black and white. A total of 81 renders (2D reconstruction images; Figure 1) were made from each 3D T2‐FLAIR MRI, simulating various camera and lighting positions. These face renders approximate black and white photos, allowing use with software packages developed for matching faces/features between photographs.

Open‐source Python packages (OpenCV, DeepFace, face_recognition, scikit‐learn) and their included pre‐trained models (designed for photographs) were used to match participant photographs to the labelled library of MRI‐based face renders. A correct match was recorded when the top‐scoring render or set of renders automatically chosen by the algorithm was from the MRI of the correct participant.

**Result:**

Feature matching with ORB and SWIFT (both from OpenCV) produced no correct matches, despite a prior publication reporting moderate success. The best match rates (Figure 2) were 58‐59% accuracy using face_recognition or DeepFace packages with support vector machines (SVMs). Without SVMs, these packages performed modestly worse (51%‐55%).

**Conclusion:**

While the highest match rates obtained by widely available open‐source packages (roughly 60%) are much lower than those of a limited‐access commercial product (Microsoft Azure, with 98%), they show that actors with only access to no‐cost readily available software could still re‐identify a research subject from a brain scan most of the time. This further highlights the need for image refacing software, such as mri_reface, to protect the privacy and identifies of research volunteers.